# Systemic Approach to Virulence Gene Network Analysis for Gaining New Insight into Cryptococcal Virulence

**DOI:** 10.3389/fmicb.2016.01652

**Published:** 2016-10-27

**Authors:** Antoni N. Malachowski, Mohamed Yosri, Goun Park, Yong-Sun Bahn, Yongqun He, Michal A. Olszewski

**Affiliations:** ^1^Division of Pulmonary and Critical Care Medicine, Department of Internal Medicine, University of Michigan Medical School, Ann ArborMI, USA; ^2^VA Ann Arbor Healthcare System Research Service (11R), Ann ArborMI, USA; ^3^The Regional Center for Mycology and Biotechnology, Al-Azhar UniversityCairo, Egypt; ^4^Department of Biotechnology, College of Life Science and Biotechnology, Yonsei UniversitySeoul, South Korea; ^5^Unit for Laboratory Animal Medicine, University of Michigan Medical School, Ann ArborMI, USA; ^6^Department of Microbiology and Immunology, University of Michigan Medical School, Ann ArborMI, USA; ^7^Center for Computational Medicine and Bioinformatics, University of Michigan Medical School, Ann ArborMI, USA

**Keywords:** systems biology and network biology, *Cryptococcus neoformans*, bioinformatics, host–pathogen interaction, curation, data collection, mouse models, virulence

## Abstract

*Cryptococcus neoformans* is pathogenic yeast, responsible for highly lethal infections in compromised patients around the globe. *C. neoformans* typically initiates infections in mammalian lung tissue and subsequently disseminates to the central nervous system where it causes significant pathologies. Virulence genes of *C. neoformans* are being characterized at an increasing rate, however, we are far from a comprehensive understanding of their roles and genetic interactions. Some of these reported virulence genes are scattered throughout different databases, while others are not yet included. This study gathered and analyzed 150 reported virulence associated factors (VAFs) of *C. neoformans*. Using the web resource STRING database, our study identified different interactions between the total VAFs and those involved specifically in lung and brain infections and identified a new strain specific virulence gene, *SHO1*, involved in the mitogen-activated protein kinase signaling pathway. As predicted by our analysis, *SHO1* expression enhanced *C. neoformans* virulence in a mouse model of pulmonary infection, contributing to enhanced non-protective immune Th2 bias and progressively enhancing fungal growth in the infected lungs. Sequence analysis indicated 77.4% (116) of total studied VAFs are soluble proteins, and 22.7% (34) are transmembrane proteins. Motifs involved in regulation and signaling such as protein kinases and transcription factors are highly enriched in *Cryptococcus* VAFs. Altogether, this study represents a pioneering effort in analysis of the virulence composite network of *C. neoformans* using a systems biology approach.

## Introduction

*Cryptococcus neoformans*, a basidiomycetous pathogenic yeast, is a leading cause of fatal mycosis in AIDS patients (Chuck and Sande, 1989) and a major cause of fungal meningoencephalitis and central nervous system (CNS)-related mortality around the world (Jarvis and Harrison, 2007). Most infections with *C. neoformans* begin in the lungs and later disseminate systemically to other organs, most critically, the CNS (Tripathi et al., 2012). *C. neoformans* is also an opportunistic pathogen in organ transplant recipients and patients with hematological malignancies (Pappas et al., 2001). Although *C. neoformans* infections are mostly a manifestation of immune deficiency, up to 25% of cases reported in the USA represent patients without recognizable immunodeficiencies (Baddley et al., 2008), and similar cases are reported in other parts of the world (Hofman et al., 2004; Zahra et al., 2004; Chen et al., 2008).

*Cryptococcus neoformans* is broadly distributed worldwide, is most frequently isolated from cryptococcosis patients with HIV/AIDS, and can be classified as serotype A, D, or their rare hybrid, serotype AD (Kwon-Chung and Bennett, 1992; Mitchell and Perfect, 1995). Cryptococcal strains display different levels of virulence, ranging from extremely virulent strains that can infect non-immunocompromised individuals to completely avirulent. These levels of virulence are associated with differential expression levels of virulence-associated genes, of which most have been identified and studied by functional genetic analysis of a mutant or using a large-scale mutant screening.

Virulence associated factors (VAFs) are an important class of gene products that help pathogens to survive within the host through adaption to specific environmental conditions found in different organs/tissues and through evasion of or interference with certain host-defense mechanisms. For example, some VAFs facilitate intracellular survival of *C. neoformans* in macrophages (Casadevall and Pirofski, 2009; Lee et al., 2010; Davis et al., 2015) while others induce pathology-related stress-response pathways in both *Cryptococcus* and the host (Fan et al., 2005; Coelho et al., 2015). VAFs allow for establishing infection, contribute to pathogenesis and thereby promote the disease state within hosts.

Thus far, many signaling pathways involved in regulating differentiation and pathogenicity of *C. neoformans* have been identified, for example, cAMP-PKA pathway, three MAP kinase pathways involving Cpk1, Hog1, and Mpk1, the Ras pathway and the calcium-calcineurin pathway (Kozubowski et al., 2009). These pathways contain numerous different VAFs. The genes encoding these VAFs were tested by various laboratories to establish their possible roles in lung and/or brain virulence using *in vivo* animal models (Alspaugh et al., 1998; Olszewski et al., 2010) or *in vitro* assays using immune cell cultures, predominantly macrophage cell lines (Rohatgi and Pirofski, 2015; Shen and Liu, 2015). Genes that affect virulence in these models are considered classical VAFs. Some of the “classical” VAFs are predicted to work on the outside of the cells inducing host-cell damage (Qiu et al., 2013), but in a broader view many VAFs work by improving fitness of the microbe, allowing it to survive in the environment within the host that is different from the external environment in terms of nutrients availability, O_2_ and CO_2_ concentrations, or the presence of host-derived anti-microbial factors (Mori, 1999).

Bioinformatics approaches have been used to increase the understanding of biological processes and development of new algorithms and statistical measures that assess relationships among members of large data sets. The rapid development of *in silico* analytical tools allows for such analysis. For instance, analysis and interpretation of nucleotide and amino acid sequences, protein domains, and protein structures enables us to create databases focusing on various levels of virulence of microbes (Xiang et al., 2007). Several recent studies used bioinformatics to identify genes responsible for fitness, genes encoding virulence traits, metabolic pathways and finding target molecules for potential vaccines against pathogenic microbes such as *Staphylococcus aureus* (Valentino et al., 2014; Delfani et al., 2015). However, although the virulence genes of *C. neoformans* and their amino acid sequences are present in various databases, we are far from a comprehensive understanding of their interactions.

Such a comprehensive analysis can be conducted using STRING. STRING database (Search Tool for the retrieval of interacting Genes) is a protein–protein interaction database analysis program generating a network of interactions from a variety of sources, including different interaction databases, text mining, genetic interactions, and shared pathway interactions (Szklarczyk et al., 2015). In addition to finding possible interactions through STRING analysis, a motif analysis can be conducted to identify all motifs in each VAF and motifs which are more commonly shared by several different VAFs. Finally, membrane localization/distribution of virulence associated genes may affect their function in pathogenicity. To determine the membrane distribution of different VAFs we used TMHMM software analysis. TMHMM utilizes a hidden Markov model for predicting transmembrane topology and identifying transmembrane proteins from soluble proteins (Krogh et al., 2001).

A previous study by Kim et al. (2015) has focused on protein–protein interactions of cryptococcal VAFs, establishing a method of predicting proteins associated with pathogenicity traits. However, STRING offers several features not present in CryptoNet (Kim et al., 2015), including the possibility to highlight functional domains, KEGG pathways, and molecular functions. We have used these features to analyze a majority of the known VAFs of *C. neoformans* for the first time in this study, in an attempt to learn something greater than the sum of the component genes.

A systemic analysis was performed to identify transmembrane VAFs with a special focus on those causing infections to the lungs and the brain. A motif analysis was conducted to identify all motifs in each VAF and motifs shared by different VAFs. Our application of these three methods of sequence-based prediction identified specific patterns in different strains of *Cryptococcus* VAFs; and apart from already known pathways, our study identified virulence function of a new strain-specific gene, *SHO1*, likely to be involved in the Hog1 mitogen-activated protein kinase (MAPK) signaling pathway.

## Materials and Methods

### Data Annotation of *C. neoformans* VAFs

**FIGURE 1:**
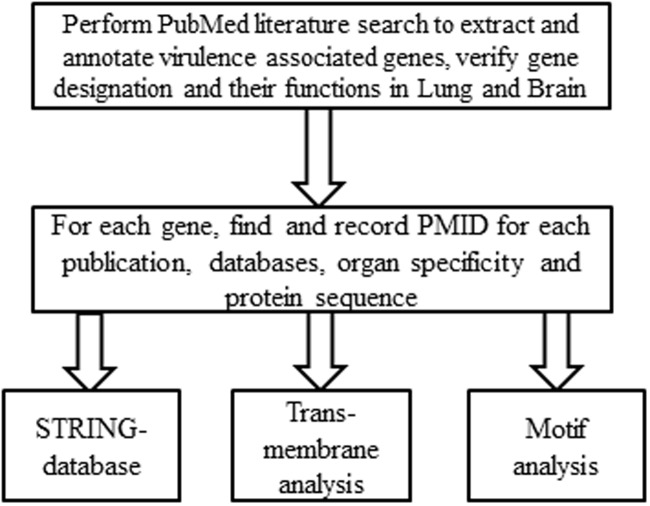
**Systemic workflow of the annotation of *Cryptococcus neoformans* virulence associated factors (VAFs).** To generate a list of VAFs and their target organs the entire literature regarding *C. neoformans* was searched for virulence studies. For every VAF found, the PMID of the corresponding peer-reviewed article was recorded, and organ specificity was deduced from the data. Gene information was then identified from the Broad Institute’s *C. neoformans* database and/or the NCBI Gene website, and the protein sequence was extracted for further analyses.

We manually collected information on 150 virulence related factors from peer-reviewed publications on PubMed Literature which have been confirmed in a mammalian model of infection (**Figure 1**). For each VAF, we found and recorded PMID(s)^1^ corresponding to a peer-reviewed article(s) that provided evidence for asserting a *Cryptococcus* protein as a VAF (**Supplementary Figure S1**). For each VAF, gene information was identified in the Broad Institute *C. neoformans* databases^2^ and/or the NCBI Gene website^3^, and its protein sequence recorded for further analyses.

### STRING Analysis

To predict interaction networks between VAFs, protein sequences of VAFs were put into the STRING database^4^. In the STRING database, the *Cryptococcus* organism was selected. In the current study, those VAFs related to brain and lung infections were used to generate interaction networks for *C. neoformans* B-3501. For generating the figures a confidence cutoff of 0.4 was used. The resulting network view, under evidence view, was downloaded for the figure. The database view contains other views of predicted functional partners, for example, neighborhood and fusion of these VAFs. To identify different VAFs involved in MAPK pathways of the pathogenic *Cryptococcus* species, the enrichment function was used, which contains various pathways found on the Kyoto Encyclopedia of Genes and Genomes.

### Transmembrane Analysis

To determine transmembrane probability for each VAF, its protein sequence was pasted to TMHMM Server v. 2.0^5^) Library in the FASTA format and analyzed using standard settings. To further determine if there would be a difference in predicted membrane location among factors associated with lung vs. brain invasiveness, a separate TMHMM analysis was performed for four groups of VAFs: lung alone, brain alone, factors involved in both brain and lung, and other factors not yet linked to lung or brain invasion. Furthermore, the annotated genome was downloaded from the Broad Institute’s Cryptococcus database and input into STRING-db. The output file was then analyzed using a Python script to log total number of transmembrane proteins and the number of transmembrane helices in each protein.

### Motif Analysis

To identify common motifs from the VAFs, protein sequences were queried against http://www.genome.jp/tools/motif/MOTIF.html Library using Pfam (determining positions and description for each VAF) as shown in **Figure 1**. Default genome.jp settings were used for this, with an *E*-value cutoff score of 1.0.

### Construction of the *sho1*Δ*::SHO1-FLAG* Complemented Strain

We used *C. neoformans* var. *grubii* wild-type (H99 strain) and the *sho1*Δ mutant strains (YSB1719) (Kim et al., 2015). The *sho1*Δ*::SHO1* complemented strain was constructed as follows. The terminator region of Sho1 was amplified via PCR by using B4796 and B4797 containing NotI, SacII restriction sites, respectively, and cloned into a plasmid pTOP-V2 (Enzynomics). The promoter and ORF region of Sho1 were amplified via PCR by using B4794 and B4795 including SalI, NotI restriction sites, respectively, and cloned into a plasmid pTOP-V2 (Enzynomics). The B4795, containing FLAG sequence (5′-CTTATCGTCGTCATCCTTGTAATC-3′). After sequencing, first the NotI and SacII digested SHO1T was subcloned into a plasmid pJAF12 containing NEO (nourseothricin) resistance marker. Second, the SalI and NotI digested insert was subcloned into the SalI and NotI digested pJAF12-SHO1T to produce a plasmid pJAF12-Sho1-FLAG. The HindIII-digested linearized pJAF12-SHO1-FLAG was transformed into the *sho1*Δ mutant (YSB1719) and targeted re-integration of the *SHO1-FLAG* gene into its native locus was confirmed by diagnostic PCR. Functionality of the *SHO1-FLAG* fusion gene in the new strain (YSB2141) was confirmed by phenotypic analysis.

### Mice

C57Bl/6 (Jackson Labs, Sacramento, CA, USA) were housed at the Veterans Affairs Ann Arbor animal care facility, and were used at around 7–9 weeks of age. At the time of data collection, mice were humanely euthanized by CO_2_ inhalation. All experiments were approved by the University Committee on the Use and Care of Animals and the Veterans Administration Institutional Animal Care and Use Committee.

### Intratracheal Inoculation of *C. neoformans*

Mice were anesthetized via intraperitoneal injection of ketamine/xylazine (100/6.8 mg/kg body weight). The trachea was exposed by making a small incision on the throat and moving aside underlying salivary glands and cutting away muscle around the trachea. 10^4^ cells of *C. neoformans* were inoculated into the lungs by injecting 30 μL of infection mix (see section above) via 30 gauge needle. After inoculation the salivary gland was moved back into place and the incision was closed using a cyanoacrylate adhesive.

### Cytokine Assays

Dissected lungs were homogenized in 2 ml of sterile water with protease inhibitor and homogenates spun at 10000 RPM for 10 min to collect supernatants that were stored frozen at -80°C until cytokines were analyzed. Quantification of levels was performed using LEGENDplex cytometric bead array (CBA) and analyzed as described previously (Xu et al., 2016). Lung associated lymph nodes were dissected and processed, as described by Xu et al. (2016). Total RNA from node cells was isolated, cDNA converted and qPCR analysis performed with data normalized to 18S mRNA, as described by Xu et al. (2016).

### CFU Assay

Lung homogenates were serially diluted in sterile water. Series of 10-fold dilutions of the samples were plated on Sabouraud dextrose agar plates in duplicates in 10-μl aliquots and incubated at room temperature. *C. neoformans* colonies were counted 2 days later, and the number of CFUs was calculated on a per whole lung basis.

## Results

### Identification of 150 of *C. neoformans* Virulence Genes through Literature Annotation

Through manual literature annotation, we identified reports on 150 *C. neoformans* genes which have an effect as established through *in vivo* mammalian infection models. This group is defined by experimental evidence of attenuation of virulence in a mouse model. For each gene on our list, the following information was annotated: (1) paper PMID; (2) protein sequence; (3) gene symbol; (4) protein sequence; and (5) for confirmed VAF’s effect on infected organ burden in various articles.

**FIGURE 2:**
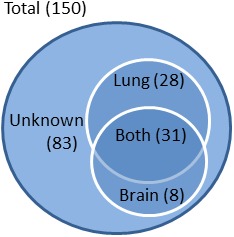
**Classification of VAFs according to target organ.** VAFs were categorized according to the organ associated with their virulence based on fungal burden assays. Of a total 150 VAFs, 28 were lung-specific, 8 were brain-specific, and 31 were associated with both lung and brain virulence. No data is available on organ specificity of 83 of the VAFs.

The 150 *C. neoformans* VAFs identified in this study were all found in STRING db10 from the total identified genes of *C. neoformans*. The network is composed of the interactions between 150 VAFs that had an impact on the survival of infected mice. Of these VAFs, 8 had documented effects in promoting brain infection, 28 were related to lung infection, 31 genes had dual effects for fungal invasiveness in both organs, and 83 affected survival with target organ partially or completely unknown (**Figure 2**). In some instances, an assay for brain-virulence was conducted without attention to cryptococcal level or pathology in the lungs, providing evidence for brain virulence but without elucidating the role of the gene in lung infection.

### STRING Protein–Protein Analysis of Virulence Genes of Different Strains of *Cryptococcus*

While CryptoNet has already been used to predict protein–protein interactions in *C. neoformans* virulence genes, STRING offers several additional features. STRING is more liberal with assigning interactions, when compared to CryptoNet, because it uses data from homologous protein interactions in other organisms. A homology comparison feature is incorporated into STRING, which makes it easier to determine the function of un-identified *C. neoformans* genes. In addition to these features which we used in this study, STRING also has other useful features, including a feature that allows for homology comparisons in a phylogenetic context, and protein family analysis.

**FIGURE 3:**
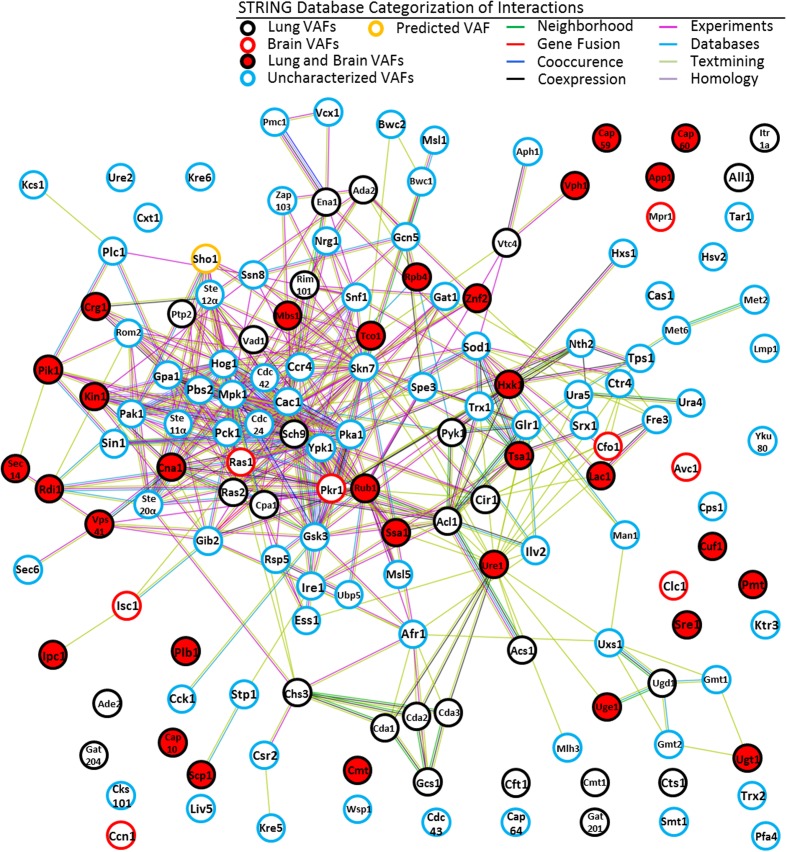
**STRING network of VAFs of *C. neoformans.*** Following annotation of 150 cryptococcal genes and establishing organ specificity for each a network was generated using STRING-db. We also included a predicted VAF, color coded in orange, to illustrate the predictive ability of STRING. The figure is color coded to indicate organ specificity of the established virulence factor and the interaction ‘strings’ are also color coded to express by what means interaction was determined, as shown in the legend. By matching the STRING ID # from the outputted image to our collection of VAFs we superimposed the organ specificities and the gene symbols of the corresponding genes. This allows any cryptologist to get an immediate idea of where a gene of interest lies relative to all other cryptococcal virulence genes within the context of informatics.

Our STRING analysis of *C. neoformans* B-3501 strain generated networks for 150 VAFs associated with lung and brain virulence. These networks include 410 interactions while 81 interactions could be expected if the collection of genes were randomly chosen among *C. neoformans* genes (**Figure 3**). This indicates that VAFs act in orchestration.

The nodes on **Figures 3** can be loosely classified into two groups – one group defined by green text-mining interactions and another group of nodes connected by interactions of all colors. The area that is defined by text-mining interactions is primarily composed of proteins involved in metabolism. The area which is densely filled with multi-colored interactions is composed primarily of transcription factors and kinases. Additionally there are many disconnected nodes, which may be loosely regulated constitutively expressed genes or genes whose interacting partners have not been discovered yet in *C. neoformans*, or proteins whose homologs have not been detected in other species by STRING.

Our STRING analysis of *C. neoformans* VAFs suggests that there are still many virulence factors to be discovered, and that not all protein interactions have been elucidated. One of the disconnected nodes in the STRING analysis, Cuf1, is a copper dependent transcription factor (Waterman et al., 2007). It is expected that functioning as a transcription factor, Cuf1 regulates transcription of genes including some genes that are involved in mediating pathogenicity. Therefore analysis of genes interacting with *CUF1* may help to identify factors that have not been yet recognized as VAFs.

Alternatively, it could mean that STRING has not yet effectively incorporated all possible protein–protein interactions from the literature. For instance, the CAP genes, which are important for synthesis of the Glucuronoxylomannan (GXM) capsule, do not yet have any functional interacting partners in STRING. It has been shown, however, that capsule expression depends on high CO2 and low-iron conditions (O’Meara and Alspaugh, 2012). This would indicate that the CAP genes are interacting partners with CO2 response and iron regulation genes *cac1* and *cir1*, or some intermediate gene. It appears that these interactions were missed by the STRING algorithm when it was mining its sources.

In addition, using the analysis functions of STRING allows us to highlight the importance of kinases and the galactoxylomannan (GalXM) capsule components. The GXM/GalXM capsule is a defining feature of *C. neoformans* infection and its prominence is highlighted using STRING. The expression of the capsule is regulated by several pathways, discussed above and below, which incorporate numerous kinases. The *CAP* genes are identified as mannosyl-transferases, crucial for creating cross-links of GXM. The GDP-mannose transporters are primarily important for export and import of the polysaccharide capsule (Cottrell et al., 2007). Finally, four (Man1, Uxs1, Ugd1, and Uge1) of the highlighted sugar metabolism proteins are involved in synthesizing the saccharide components of the capsule.

Additional information about each protein from our database cannot be described in detail here, but can be gained by interactive viewing of our STRING network at^6^ when our database (**Supplementary Table S1**) is imported.

### Analysis of VAFs in the MAPK Pathway using STRING

**FIGURE 4:**
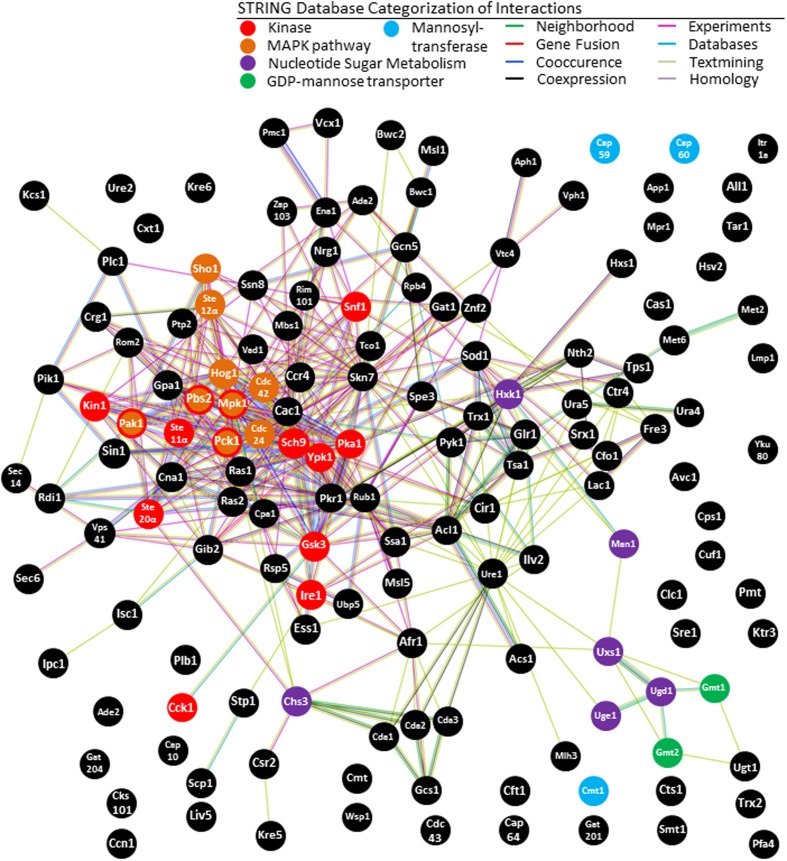
**KEGG analysis of STRING network of *C. neoformans*.** Our study underlines the importance of the mitogen-activated protein kinase (MAPK) pathway in the virulence network of *C. neoformans*. STRING analysis also shows enrichment of galactoxylomannan synthesis and transport, supporting previous findings regarding the importance of this capsule saccharide. The central role of kinases in the *C. neoformans* virulence network is also underlined, as is expected from an opportunistic pathogen that requires rapid upregulation of virulence factors upon entry into the host.

Using STRING analysis we are able to illustrate a key kinase pathway that is central to the *C. neoformans* virulence network (**Figure 4**). Hog1 (located in the center of the interaction chart) is a MAP kinase involved in a signal transduction pathway that is activated by changes in the osmolarity in the extracellular environment (Bahn et al., 2005). Hog1 controls osmotic regulation of transcription of target genes. Hog1 is also involved in response to UV radiation and mediates sensitivity to fludioxonil. Hog1 has several strong predicted functional interactions with: Ste7 (a MAP kinase modulating capsule formation; Feretzaki et al., 2014), Sch9 (a protein kinase Sch9 modulating cell wall formation; Wang et al., 2004), Gpa1 (a G-protein alpha subunit homolog required for melanin synthesis, capsule formation and other functions) (Alspaugh et al., 1997; O’Meara et al., 2010a), and Ras1 (Ras superfamily GTPase that controls thermotolerance) (Waugh et al., 2002; Ballou et al., 2013). The most commonly reported biological functions associated with these proteins are linked to regulation of *C. neoformans* pathogenicity, although they appear to regulate many other aspects of fungal life cycle.

Analysis of total VAFs using Kyoto Encyclopedia of Genes and Genomics (KEGG) tools in STRING-db indicates that the MAPK pathway is the most prominent among virulence factors. Eight VAFs constitute the MAPK signaling pathway with (*p* = 8.6 e^-4^). The proteins are, Hog1, Ste12alphap, MPK1, Pbs2, Pak1, Pck1, CDC42, and CDC24. The KEGG is a collection of databases dealing with genomes, biological pathways, diseases, drugs, and chemical substances, and has been incorporated into the analysis function of STRING (Kanehisa and Goto, 2000). KEGG has been widely utilized for bioinformatics research and education, including data analysis in genomics, metagenomics, metabolomics, and other -omics studies, modeling and simulation in systems biology, and translational research in drug development (Kanehisa et al., 2010).

**FIGURE 5:**
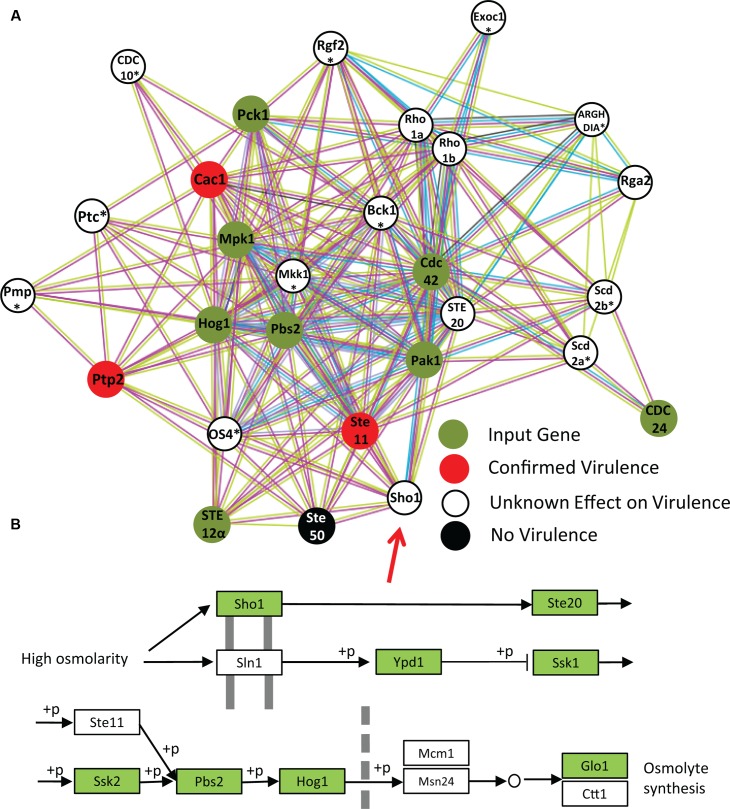
**Network analysis of MAPK pathway suggests involvement of Sho1 in virulence.** In order to test STRING as a predictor of new virulence factors, all identified VAFs from the MAPK pathway were inserted into STRING to select the top 20 interactors **(A)**. The identity of proteins marked with an asterisk (^∗^) could only be obtained from homologous proteins. One of the top predicted partners is Sho1. The role of Sho1 in *C. neoformans* MAPK pathway has not been investigated until very recently, perhaps because it is absent in the laboratory strain JEC21. However, we have confirmed its presence in the *C. neoformans* B-3501 genome by STRING analysis and also the KEGG **(B)**.

In order to more closely investigate this interaction within MAPK gene network the eight regulators of the MAPK pathway were inserted into STRING-database (**Figure 5A**). The top 20 predicted interacting partners include several genes that are confirmed virulence factors, and many genes which have not yet been investigated in the context of virulence. Among the four functional partners in this group that have already been tested, three are confirmed to be VAFs *in vivo* at the time of publishing. Most have not yet been named or studied in *C. neoformans*, however. Of the predicted VAFs that have not been tested *in vivo* for virulence (**Figure 5A**) only three have been knocked out in: *RHO. STE20*, and *SHO1*. However, *RHO* has three close homologs, and thus determination of its role in pathogenesis will require extensive studies with many combinations of knockouts. On the other hand, *SHO1* is a gene already known to be a protein upstream of the MAPK pathway in another pathogenic fungus *Candida albicans*, where it serves as cell membrane-bound receptor protein sensing external osmolality (Biswas et al., 2007). *STE20* is also a good candidate for study, however, it is down-stream from *SHO1* in the HOG pathway (**Figure 5B**). Considering all these factors, *SHO1* was selected as the best gene target for verification of virulence in *C. neoformans* infection model.

### Role of Sho1 in *Cryptococcus neoformans* Pathology

Initial studies lead to the conclusion that *Sho1* was absent in *C. neoformans*. Analysis using STRING and using the KEGG indicated that Sho1 is present in serotype A strain H99 and the serotype D strain B-3501 (**Figure 5B**), but not present in the serotype D strain JEC21. We suspect that this was the reason why a *Sho1* homolog has not been detected in *C. neoformans* until 2015, when it was characterized in H99 as potential factor in virulence pathways by another bioinformatics study. Kim et al. (2015) conducted an informatics-based genome-wide study to identify novel genes for virulence and adaptation to chemical stresses, pinpointing Sho1 among other VAF candidates. Their study, using A/J mice, has not shown significant effect of Sho1 on the survival rate of infected mice. Due to the prominence of this gene in, and in relation to other genes upstream of, the important HOG pathway, we decided to conduct a study of fungal burdens in an experimental mouse model of cryptococcosis to possibly determine Sho1 role as a factor associated with fungal growth in the lungs.

A common mechanism by which cryptococcal virulence factors confer better survival of the pathogen in the host is through interference with either innate or adaptive host immune response or both (Olszewski et al., 2010). We analyzed fungal burdens in the infected lungs of C57BL/6 mice at time points consistent with the immediate adaptation to the host tissue conditions (day 3), interference with the innate (day 7), and with the adaptive phase of the immune response (day 14). Lungs were collected and homogenized to assay for fungal burdens. Cytokine response was also assessed for possible effects of Sho1 on Th1/ Th2 bias, which is an important index of protective vs. non-protective response used in *C. neoformans* virulence studies (Osterholzer et al., 2009; Zhang et al., 2010; He et al., 2012; Qiu J. et al., 2012).

**FIGURE 6:**
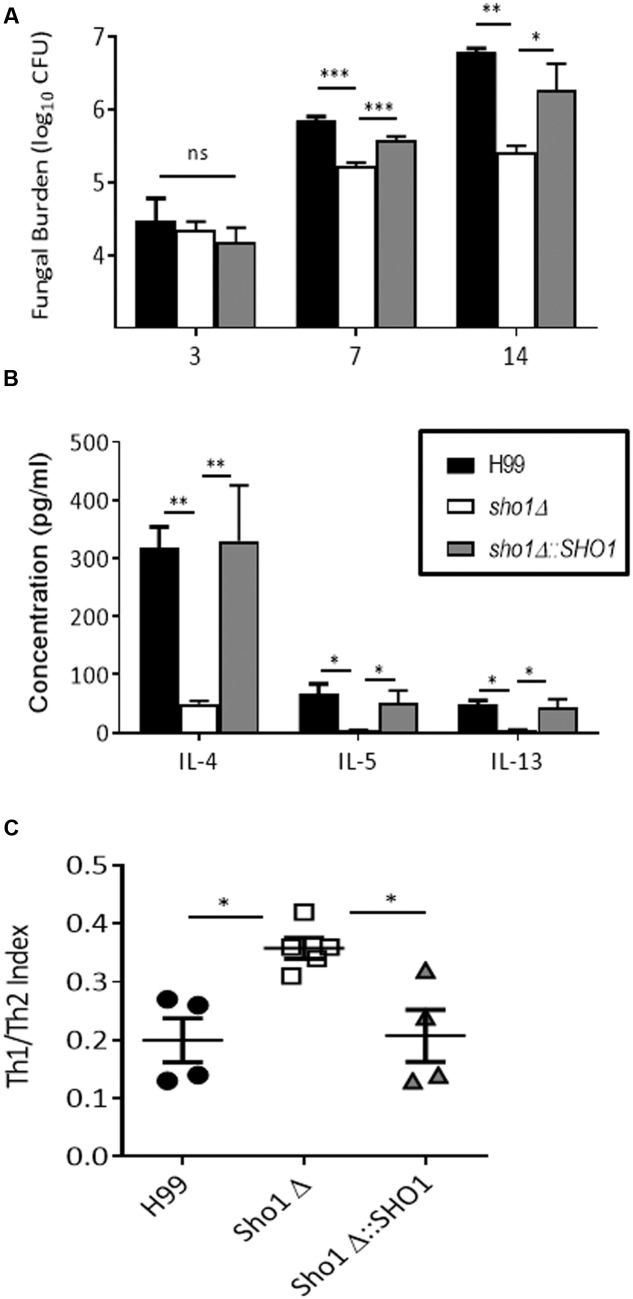
**Sho1 enhances pulmonary virulence of *C. neoformans* by skewing lung cytokine balance toward non-protective Th2 profile.** C57Bl/6 mice infected intratracheally with 10^4^ cells of wild-type strain H99 *C. neoformans. SHO1*-deleted mutant *sho1*Δ, or complement strain *sho1*Δ*::SHO1* and analyzed at selected time points. Lungs were homogenized, serially diluted and plated for fungal burden evaluation **(A)**. Cytokine levels in supernatant from undiluted homogenates were evaluated by CBA **(B,C)**. Significant differences between fungal burdens in the lungs infected with *sho1*Δ vs. those infected with *SHO1*-sufficient strains were found at days 7 and 14, but not day 3 post-infection, indicating that *SHO1* is not required for initial survival of organism in the host, but is important for pulmonary virulence **(A)**. Furthermore, non-protective Th2 cytokines IL-4, IL-5, and IL-13 were highly abundant in the lungs infected with *SHO1*-sufficent strains and only minimally induced in those infected with *sho1*Δ **(B)**. The overall Th1/Th2 cytokine balance was expressed as Th1/Th2 index ratio at day 14. Ratio of summarized Th1 cytokines (IFNγ and TNFα) to Th2 cytokines (IL-4, IL-5, and IL-13) shows that cryptococcal *SHO1* deletion resulted in a shift toward protective Th1 and away from non-protective Th2 cytokines. Parametric data were analyzed by ANOVA with Dunnet’s post-test while ratios were analyzed by Kruskal–Wallis non-parametric test. *N* = 4 or above mice per time point per group ^∗^*p* < 0.05, ^∗∗^*p* < 0.01, ^∗∗∗^*p* < 0.001.

This fungal burden assay indicated no significant difference between wild type strain and the *sho1*Δ and *sho1*Δ*::SHO1* mutants on day 3, but significantly lower fungal burdens in the *sho1*Δ-infected lungs were detected on days 7 and 14 compared to wild type and *sho1*Δ*::SHO1* infections (**Figure 6A**). These results indicate that *SHO1* expression is not required for the immediate adaptation of *C. neoformans* to lung environment but it supports optimal growth rate of the pathogen at time points consistent with the innate fungal control (Eastman et al., 2015) and the development of the polarized adaptive immunity (Osterholzer et al., 2009; Eastman et al., 2015), suggesting that *SHO1* may have a role in promoting immunomodulatory effects of *C. neoformans*.

In support of this, we observed greatly enhanced levels of non-protective Th2 cytokines (IL-4, IL-5, and IL-13) in lungs infected with *SHO1*-sufficient strains compared to lungs infected with *sho1*Δ at day 14 (**Figure 6B**). Since the much higher fungal burdens in H99 and *sho1*Δ*::SHO1* at day 14 likely contributed to overall higher cytokine levels in these groups, we analyzed Th1 (IFNγ and TNFα) vs. Th2 (IL-4, IL-5, and IL-13) cytokine ratios generating combined Th1/Th2 cytokine index ratio (**Figure 6C**). This index reflects overall balance of these cytokines in each infected animal, rather than individual cytokine levels and thus is much less affected by the fungal load. This analysis revealed that the Th1/Th2 cytokine balance was consistently and significantly more skewed toward Th1 in *sho1*Δ-infected lungs and more toward Th2 in the lungs infected with *SHO1*-sufficient strains (**Figure 6C**), suggesting that cryptococcal *SHO1*-expression contributes to the development of non-protective Th2 bias.

To further support this we assessed gene expression in lung associated nodes on day 7, a time point consistent with early priming of the adaptive immune response in the nodes (Xu et al., 2016). Accordingly, we have found that *SHO1*-deletion enhanced early expression of protective cytokines IFNγ and IL-17 (**Supplementary Figure S1**) and resulted in strong trends in upregulation of transcription factors Tbet and RorγT, responsible for the development of protective Th1 and Th17 cells. Thus, cryptococcal *SHO1* expression clearly promoted expansion of *C. neoformans* in the infected lungs and contributed to immunomodulatory potential of *C. neoformans.*

### Transmembrane Analysis of Virulence-Associated Genes of *C. neoformans*

Both soluble and transmembrane proteins should be important for the pathogenicity of *C. neoformans*; however, these proteins have not been systematically studied as a group. Multiple *in vivo* studies confirm that intracellular proteins involved in metabolism and catabolism are vital to *C. neoformans* proliferation in the lungs and dissemination to the brain (Olszewski et al., 2004; Qiu Y. et al., 2012). On the other hand, the outer capsule is considered one of the defining pathogenicity factors of *C. neoformans*. Acapsular strains of *C. neoformans* are generally categorized as non-pathogenic (Okabayashi et al., 2005; O’Meara and Alspaugh, 2012). Also, many studies indicate membrane composition in *C. neoformans* determines various aspects of pathogenicity, for instance ability to cross the blood brain barrier, and organization of VAFs on the fungal membrane (Siafakas et al., 2006; Singh et al., 2012; Stie and Fox, 2012). Here we classify our collection of VAFs into soluble and transmembrane VAFs using TMHMM.

**FIGURE 7:**
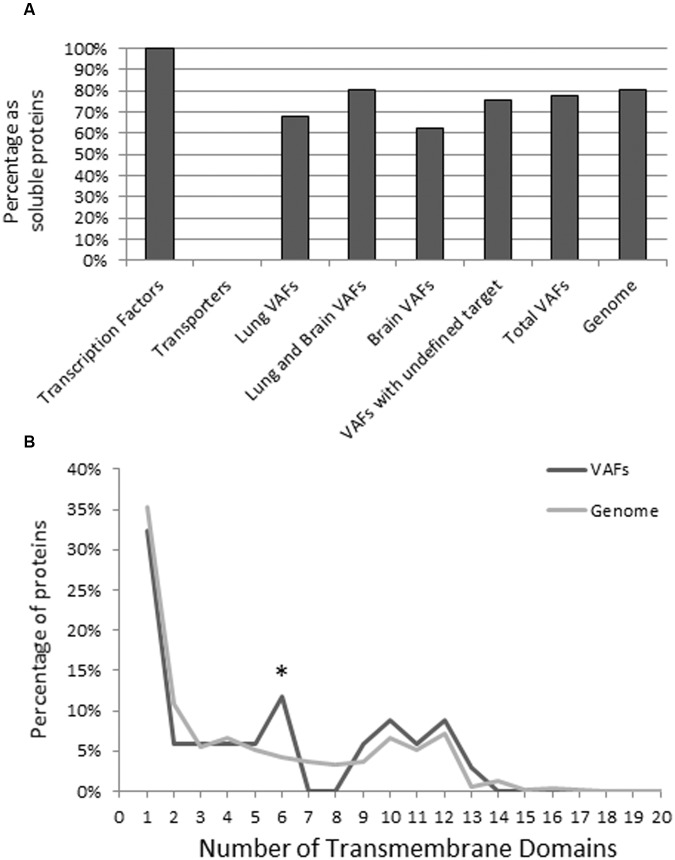
**Transmembrane analysis of known *C. neoformans* virulence factors compared to all annotated *C. neoformans* genes.** Protein sequences were input into TMHMM Server V 2.0, and the resulting plot was interpreted to determine membrane specificity (transmembrane or soluble) of the virulence factor. Transcription factor and transporters are included as a negative and positive control, respectively **(A)**. To look at the composition of transmembrane proteins we also analyzed the number of transmembrane helices of each transmembrane protein. The maximum number of transmembrane helices was 20, but only proteins with less than 15 transmembrane helices were frequent. This study indicates that compared to the genome, VAFs are enriched in proteins with six transmembrane helices but otherwise have a similar distribution of the number of transmembrane helices **(B)**. Data were analyzed using Pearson’s chi-square test. ^∗^*p* < 0.05.

TMHMM v 2.0 utilizes a hidden Markov model to determine transmembrane domains on proteins, with the ability to distinguish cytoplasmic and outer domains (Krogh et al., 2001), and is currently one of the most accurate membrane protein topology prediction methods. To validate the accuracy of TMHMM, we tested the transporters and transcription factor VAFs of *C. neoformans*. Because transporters are inherently membrane-associated and transcription factors localize in the nucleus, these are positive and negative controls, respectively (**Figure 7A**).

Overall, the majority of VAFs showed characteristics of soluble proteins regardless of their association with target organs. Using the TMHMM method, we found that of the 150 known organ specific VAFs of *C. neoformans* B-3501 77.3% (116) are soluble VAFs and 22.7% (34) are transmembrane (**Figure 7A**). Among CNS and lung-virulence-related VAFs (31): 81% (26) were soluble and 19% (9) were membrane associated. Among VAFs related to pulmonary infection (28): 68% (19) were soluble proteins, 32% (9) are transmembrane proteins. The VAFs which promote infection in brain (8): consisted of 63% (5) soluble proteins and 37% (3) transmembrane proteins. Finally, among VAFs which have incompletely known target organ (83): 76% (63) were non-membrane proteins and 24% (20) were transmembrane proteins.

Additionally, we conducted a TMHMM study of all annotated *C. neoformans* genes to elucidate whether VAFs were enriched in transmembrane domains. Of a total 6962 genes, 1354 (19.4%) contain transmembrane domains, compared to 22.6% in our VAF database (**Figure 7B**). This suggests that both transmembrane and soluble proteins are equally important in the pathogenicity of *C. neoformans*.

In order to define the functionality of transmembrane VAFs we recorded their functions (**Table 1**). The largest group is ion sensors and transporters, which play a key role in regulating ion homeostasis and expression of virulence genes within the host (Silva et al., 2011; Jung et al., 2012). Proteins related to mannose and xylose transport and crosslinking are also numerous and these are the proteins required for construction of the immune-modulating capsule of *C. neoformans*. Another important group of transmembrane VAFs are involved in lipid metabolism, particularly sterols and sphingolipids, which are an important multi-functional membrane component of this fungal pathogen (Siafakas et al., 2006).

**Table 1:** Classification of transmembrane virulence associated factors (VAFs) of *Cryptococcus neoformans.*

Functional group	Number of proteins in group	List of VAF symbols
Capsule synthesis and maintenance	7	Cap10, Cap64, Cas1, Cxt1, Kre6, Ktr3, Ugt1
Miscellaneous	4	Afr1, Ena1, Liv5, Vtc4
Calcium homeostasis	3	Clc1, Pmc1, Vcx1
Cell wall	3	Chs3, Cps1, Pmt
Copper homeostasis	3	Cmt1, Ctr4, Vph1
Glycosylation/palmitoylation	3	Gmt1, Gmt2, Pfa4
Sphingolipid metabolism	3	Gcs1, Ipc1, Isc1
Sterol metabolism	3	Scp1, Smt1, Stp1
Iron homeostasis	2	Cfo1, Fre3
Nutrient transport	2	Hxs1, Itr1a


To further analyze transmembrane VAFs as a group the number of transmembrane helices present in each protein was determined, in the genome and on our list of VAFs. Although our study indicates that proteins with six transmembrane domains are more frequent among VAFs than the general gene pool (**Figure 7B**), the proteins in this group function independently of one another and do not possess functional interacting partners in STRING, so we are assuming this is only a coincidence. Although not significant at the statistical level, no VAFs with seven or eight transmembrane domains have been found. In conclusion, the proportion of transmembrane helices in VAFs is similar to that in total genomic proteins.

### Motif Analysis of *Cryptococcus* VAFs

To further expand our analysis and sort the VAFs based on their expression of functional domains, all 150 organ-specific VAFs were queried against the Motif Search Library (Kanehisa, 1997). **Table 2** lists the top common motifs in VAFs of *Cryptococcus* resulting from this analysis.

**Table 2:** Most frequent Motifs among *C. neoformans* VAFs.

Motifs symbols	Description	List of VAF symbols
Pkinase	PF00069, Protein kinase domain	14: Cck1, Gsk3, Hog1, Ire1, **Kin1**, Mpk1, Pak1, Pbs2, Pck1, Pka1, Sch9, Snf1, Ste20a, Ypk1
Pkinase_Tyr	PF07714, Protein tyrosine kinase	14: Cck1, Gsk3, Hog1, Ire1, **Kin1**, Mpk1, Pak1, Pbs2, Pck1, Pka1, Sch9, Snf1, Ste20a, Ypk1
Kinase-like	PF14531, Kinase-like	11: Gsk3, Hog1, **Kin1**, Pak1, Pbs2, Pck1, Pka1, Sch9, Snf1, Ste20a, Ypk1
APH	PF01636, Phosphotransferase enzyme family	10: Gpa1, Gsk3, Ire1, **Kin1**, Mpk1, Pak1, Pbs2, Pka1, Sch9, Ste20a
Kdo	PF06293, Lipopolysaccharide kinase (Kdo/WaaP) family	10: Gsk3, Pak1, Sch9, Snf1, Ste20a, Ypk1, Hog1, **Ipc1**, **Kin1**, Mpk1
GATA	PF00320, GATA zinc finger called zinc-containing domains	5: Bwc2, Cir1, Gat1, Gat201, Gat204
zf-C2H2	PF00096, Zinc finger, C2H2 type	5: Nrg1, Rim101, Ste12alphap, Zap103, Znf2
zf-H2C2_2	PF13465, Zinc-finger double domain	5: Nrg1, Rim101, Ste12alphap, Zap103, Znf2
zf-C2H2_4	PF13894, C2H2-type zinc finger	5: Nrg1, Rim101, Ste12alphap, Zap103, Znf2


*Motif names were grouped since they shared in the same VAF. A complete table of *C. neoformans* virulence associated factor (VAF) Motifs, categorized by organ specificity is available in the supplement. (Underline: lung, bold: brain, bold+underline: both, Non: Unknown target organ)*

The protein kinase (PK) family motif (PF00069) is present in 14 VAFs and is the most common motif among all VAFs. Since *C. neoformans* is an opportunistic pathogen, it requires efficient upregulation of pathogenicity genes once it enters the stressful host microenvironment, a role partially filled by kinase phosphorylation. Many VAFs from this pathway have been identified, for example, Tyrosine PK, Kinase-like proteins, APH (Phosphotransferase enzyme family) present in MAP kinase pathway, and cAMP-PKA pathway.

Concurrent with the high frequency of kinases, DNA binding proteins are also very frequent among VAFs. Two distinct groups of zinc fingers are found in the VAFs of *C. neoformans*: GATA and the C2H2, H2C2_2, H2C2_4 proteins. Most of these DNA binding proteins are transcription factors, which is consistent with importance of kinases as regulators in virulence. Efficient upregulation of pathogenicity factors and stress response genes is key to *C. neoformans* success in evading host immune responses. This might suggest that disrupting the fine-tuned regulatory process in *C. neoformans* may be a viable alternative to antibiotic treatments.

Finally, we analyzed a less frequent but equally interesting set of genes linked by one motif: the FoxP coiled coil domain, found in three proteins of *C. neoformans* (Znf2, Zap103, and Rim101), all of which decrease virulence in a mouse model of infection. Although the FoxP domain in mammalian leukocytes facilitates dimerization of FoxP transcription factors and commitment to a T regulatory lineage, Znf2 and Rim101 have very disparate roles and different impacts on cell morphology (Lin et al., 2010; O’Meara et al., 2010b), so it seems unlikely that they dimerize. Zap103 was only recently shown to have impact on virulence in a mouse survival screen (Kim et al., 2015); knockout of Zap103 also leads to increased virulence. The immediate question is whether the FoxP-cc domain is the reason these three proteins are all negative regulators of virulence, or whether it is just an unexpected coincidence. Additionally, as an input for STRING-db, these genes are very effective predictors of other VAFs. Of the top 20 interactors, 9 have a confirmed direct impact on organ fungal burden or survival, while 11 have not yet been tested.

## Conclusion and Discussion

In this study, we have made the first attempt to collect and analyze the total VAFs of *C. neoformans*. All VAFs studied were manually selected from the literature based solely on experimental evidence and systematically analyzed using three bioinformatics methods. We defined comprehensive interactions among these VAFs based on their reported roles in different pathogenicity pathways, as well as their involvement in pathogenesis of both lungs and the CNS infections. These identified VAFs were used to create a network using the STRING database in order to identify their relation to other genes. A systematic analysis was performed on the transmembrane proteins of this list of VAFs, comparing it to the general gene pool of *C. neoformans*.

It is an important observation that 77.3% of VAFs are soluble proteins. However, this percentage may differ according to target organ, among CNS 62% of VAF were soluble while in lung infection 68% were soluble. Our genomic TMHMM study indicated 80.4% of all annotated *C. neoformans* genes contain transmembrane domains, but this is not significantly different from the list of VAFs collected in this study. The TMHMM study also points to transporters and channels as particularly important or at least well-represented among pathogenicity factors. However, while future studies may indicate whether transmembrane proteins or transporters are in fact particularly important for cryptococcal tropism to specific mammalian organs or tissues, at this time no such trend exists.

Our systematic literature study also draws attention to the significance of a clinically important osmosensor in the high osmolarity glycerol (HOG) pathway, Sho1. While initial studies have shown no effect of Sho1 on virulence (Kim et al., 2015), our new data motivated by the bioinformatics analysis shows that Sho1 is in fact an important component of pulmonary virulence in *C. neoformans* serotype A. No difference in CFU numbers between *sho1*Δ and H99 at day 3 indicating, that Sho1 is not required for H99 survival in the naive lung environment; however, its deletion resulted in growth to lower titers compared to the *SHO1*-sufficient strains by day 7 and progressive widening of the gap in fungal burdens between *sho1*Δ and *SHO1-*sufficient strains on day 14, suggesting that Sho1 contributes to immunomodulatory properties of *C. neoformans*. Our data support this view, at least with regards to Sho1 contributing to the development of non-protective Th2 bias, which is known mechanism of cryptococcal virulence (Zhang et al., 2009; Olszewski et al., 2010). While future studies will be needed to pinpoint the exact role of Sho1 in pulmonary virulence, this analysis identified yet unknown role of cryptococcal Sho1 in promoting fungal expansion in the infected lungs and its contribution to immunomodulatory potential of the cryptococcal organism.

The absence of Sho1 in the less virulent strain JEC21 may highlight the genetic reason for the divergence of the role of the HOG pathway in cryptococcal strains JEC21 and B-3501. If Sho1 is indeed an upstream member of the HOG pathway in *C. neoformans*, like it is in *S. cerevisiae*, work relating to other MAPK pathways in *C. neoformans* may have to be re-analyzed. Two authors of this paper are currently working to specify the role of Sho1 in this context.

This study has also identified a number of motifs present in many VAFs in *Cryptococcus* by identification of domains that occur within proteins and therefore give insight into their function. This provides us a way to search these motifs (e.g., p-kinase and GATA) for development of new drugs and vaccines against cryptococcal infection. Identifying essential specific fungal genes and motifs not present in the host will enable us to design specific, non-toxic inhibitors and reveals a lot about the virulence pathways of a pathogen, providing motivation for further studies using new VAFs that become known in the future. The prominence of regulatory networks among *C. neoformans* genes tested for virulence might suggest that disrupting these networks can be an effective alternative to existing antifungal antibiotics.

Regarding future directions, our downloadable database (Supplementary Data) can be expanded either manually by adding new virulence factors as new publications become available in literature, or through introduction of algorithms that would detect automatically new reports on virulence factors. At current state of knowledge, manual annotation appears to be a superior alternative to automated annotation, especially that we are not aware of an algorithm for determining the target of study of a scientific publication at the precision level that this type of analysis requires. While this means our study may not cover 100% of proven VAFs even at the time of publishing, it still allows for a systems-level analysis of the majority of confirmed VAFs. Furthermore, following necessary updates this approach can be reused to obtain continuously more comprehensive type of analysis that may result in identification of new virulence factors and help us to understand their mutual interactions at an increasingly advanced level. We also believe that future studies combining analysis of multiple putative novel cryptococcal VAF genes in experimental infections will be needed to further demonstrate power of broader usefulness of the *in silico* analysis in discovery of new VAFs.

In summary, the results in this report are significant and novel as such comprehensive analysis to our knowledge has not been yet reported for *C. neoformans*. This detailed analysis of experimentally verified VAFs of various strains of *Cryptococcus* provides a powerful way to analyze and predict specific interactions between different VAFs and can be continuously expanded as new reports of VAFs become available. Our analysis, while making the first step in systemic studies of *C. neoformans*, constitutes a foundation for future studies. We anticipate that other researchers will be able to utilize our database and conduct bioinformatics studies of their own expanded with additional new factors. The usefulness of these systemic studies can be seen immediately, as is shown by our identification of the *SHO1* gene in the mitogen activated protein kinase pathway of *Cryptococcus* as a potential VAF and providing novel evidence that it can play a significant role in cryptococcal virulence.

## Author Contributions

AM and MY gathered and analyzed data and wrote portions of the manuscript under guidance of YH and MO, who overseen this work and edited manuscript. GP and Y-SB generated *SHO1* mutant and complement strains. Y-SB also contributed to edits of the manuscript.

## Conflict of Interest Statement

The authors declare that the research was conducted in the absence of any commercial or financial relationships that could be construed as a potential conflict of interest.

## Footnotes

1 http://www.ncbi.nlm.nih.gov/pubmed

2 http://www.broadinstitute.org/annotation/genome/_neoformans/MultiHome.html

3 http://www.ncbi.nlm.nih.gov/gene/

4 http://string-db.org

5 http://www.cbs.dtu.dk/services/TMHMM/

6 http://string-db.org
