# Supplementary figures and images for: Systemic Approach to Virulence Gene Network Analysis for Gaining New Insight into Cryptococcal Virulence
Source: Front Microbiol. 2016 Oct 27;7:1652. doi: 10.3389/fmicb.2016.01652 (PMC5081415; doi:10.3389/fmicb.2016.01652)

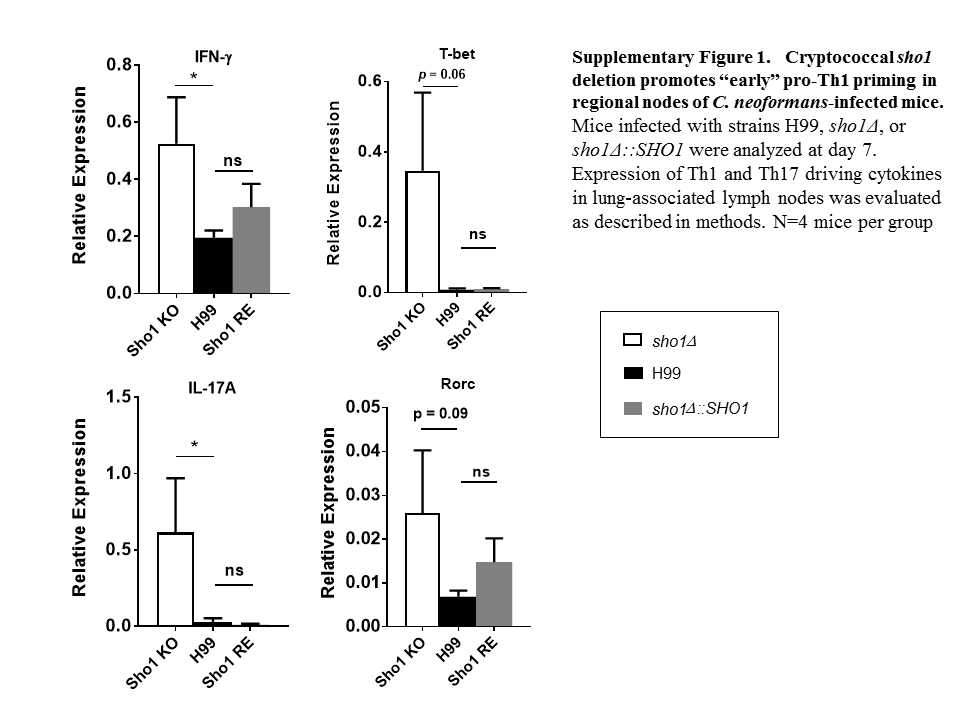

Supplement: Supplementary file 2 [file Image_1.TIF]
